# Tuberculosis Mortality and Living Conditions in Bern, Switzerland, 1856-1950

**DOI:** 10.1371/journal.pone.0149195

**Published:** 2016-02-16

**Authors:** Kathrin Zürcher, Marie Ballif, Marcel Zwahlen, Hans L. Rieder, Matthias Egger, Lukas Fenner

**Affiliations:** 1 Institute of Social and Preventive Medicine, University of Bern, Bern, Switzerland; 2 Swiss Tropical and Public Health Institute, Basel, Switzerland; 3 University of Basel, Basel, Switzerland; 4 Epidemiology, Biostatistics and Prevention Institute, University of Zürich, Zürich, Switzerland; Johns Hopkins Bloomberg School of Public Health, UNITED STATES

## Abstract

**Background:**

Tuberculosis (TB) is a poverty-related disease that is associated with poor living conditions. We studied TB mortality and living conditions in Bern between 1856 and 1950.

**Methods:**

We analysed cause-specific mortality based on mortality registers certified by autopsies, and public health reports 1856 to 1950 from the city council of Bern.

**Results:**

TB mortality was higher in the Black Quarter (550 per 100,000) and in the city centre (327 per 100,000), compared to the outskirts (209 per 100,000 in 1911–1915). TB mortality correlated positively with the number of persons per room (r = 0.69, p = 0.026), the percentage of rooms without sunlight (r = 0.72, p = 0.020), and negatively with the number of windows per apartment (r = -0.79, p = 0.007). TB mortality decreased 10-fold from 330 per 100,000 in 1856 to 33 per 100,000 in 1950, as housing conditions improved, indoor crowding decreased, and open-air schools, sanatoria, systematic tuberculin skin testing of school children and chest radiography screening were introduced.

**Conclusions:**

Improved living conditions and public health measures may have contributed to the massive decline of the TB epidemic in the city of Bern even before effective antibiotic treatment became finally available in the 1950s.

## Introduction

One hundred years ago tuberculosis (TB) was the leading cause of death from infectious diseases worldwide [1, 2]. In 2014, TB was still the second leading cause of death from an infectious disease, with over 95% of TB deaths occurring in low- to middle-income countries. TB was and is a poverty associated disease related with poor living and working conditions, including overcrowding and inadequate ventilation [2].

TB mortality data indicate that the epidemic in Europe lasted for centuries, but may have peaked as early as 1750 in the United Kingdom, where industrialisation started [3, 4]. In continental European countries, the TB epidemic probably peaked later, in the first half of the 19^th^ century [4]. At the peak, about 20–25% of adult deaths in all European countries were caused by TB [5, 6]. The decline in TB mortality over the last two centuries has been attributed to many factors, including improved social conditions and nutrition, less overcrowding, barring those with infectious TB from the workplace, and the establishment of TB sanatoria [3, 4, 7–9]. TB was recognized as the leading cause of death in Switzerland during the 19^th^ century [10, 11]. In the capital city of Bern, TB caused 15% of all deaths in 1856 [10]. At the time, Bern was one of the largest cities in Switzerland, and socioeconomic classes varied widely between its residential quarters [12]. The city council kept detailed mortality registers and public health reports, which make it an ideal site for a historic study of conditions that may have influenced the course of the TB epidemic [10]. We studied TB mortality and living conditions in Bern between 1856 and 1950.

## Methods

### Data Collection and Definitions

We identified TB-related data in mortality registers, public health and statistical reports from 1856 to 1950, available at the city council of Bern, Switzerland [10, 13–16]. Mortality data were not available for years 1868, 1869, 1871, and 1926–1928; TB mortality data stratified by age and sex were available between 1871 and 1925 [10, 15, 17]. Between 1876–1921 mortality registries included ten causes of death, expanded to 21 after 1921. These causes were established through official autopsies [10]. We classified causes as injuries (including homicide), communicable diseases (including all infectious diseases and infant death), and non-communicable diseases (including cancer). We also collected data specifically on typhoid fever mortality and infant mortality [10, 13–16].

We extracted data on living conditions in Bern between 1856–1950 from a doctoral thesis [18], and a survey that collected data on the living conditions in the different city quarters in 1896 [19]. The surveys covered 3,400 houses and 10,600 apartments. The city of Bern consisted of five quarters in the City centre (named after colours since the Napoleonic occupation), and five quarters in the outskirts. The Black Quarter was the quarter with the worst housing conditions (S1 Fig), where mainly people from the working class lived [10].

For comparison, we extracted TB mortality data for Switzerland (1877–1950) and for the city of Zürich (1830–1933) from an online database [20]. We also obtained TB mortality data from published literature: England and Wales (1851–1923) [21], Germany (1892–1950) [1], and New York (1881–1950) [22, 23].

### Statistical Analyses

We expressed mortality rates as deaths per 100,000 population and infant mortality as deaths in the first year of life per 1,000 live births. We compared living conditions between the Black Quarter (one quarter), the city centre (four quarters), and the city outskirts (five quarters), based on ecological measurements from the housing survey in 1896. We calculated the median and the range (minimum-maximum) for the city centre and the city outskirts. We used the Chi-square or Wilcoxon rank-sum test to assess differences between groups. Correlations between TB mortality (1911–1915) and living conditions (housing survey from 1896) were presented as Pearson correlation coefficients, with 95% confidence intervals. We used linear regression to model declines in TB mortality from different cities and countries. Declines in TB mortality were presented as annual decline in percentages, and the curve fit as R squared. All analyses were performed in Stata version 14 (Texas, USA).

### Geographical Analyses

We georeferenced a historic map obtained from the city council, which showed the geographic residency of TB deaths in Bern from 1920–1935. We used the QGIs 2.8 software and the CH1903/LVD03 coordinate system as the reference [24].

## Results

### Mortality Due to TB and Other Causes in the City of Bern

**Fig 1:**
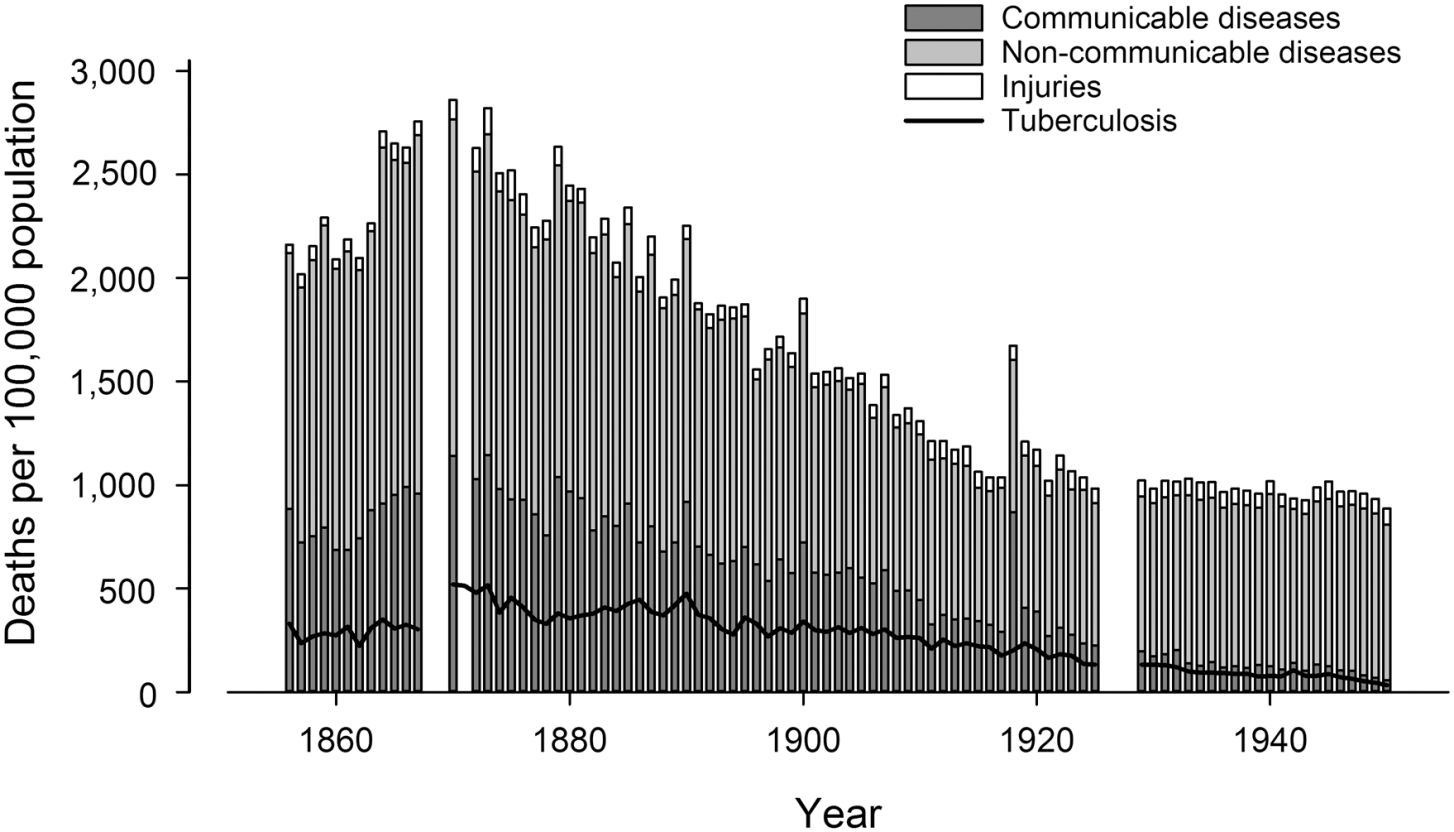
Changes in the mortality due to injuries (including homicide), communicable (infectious diseases and infant death) and non-communicable diseases (including cancer) in the capital city of Bern, Switzerland, between1856 and 1950. Bars represent average 5-year mortality; the black curve represents tuberculosis (TB) mortality. Data were not available for the years 1868–1869, 1871 and 1926 to1928.

Overall crude mortality in Bern decreased from 2,160 per 100,000 in 1856 to 888 per 100,000 in 1950 (Fig 1), with a pronounced mortality peak during the influenza epidemic in 1918. Over the same period, the population of Bern increased from 29,670 to 146,700 residents. TB mortality rates decreased 10-fold, from 330 to 33 per 100,000 (Fig 1), and typhoid mortality from 78 to 0 per 100,000 population (S2 Fig). Infant mortality declined from 208 in 1881 to 25 per 1,000 live births in 1950 (S2 Fig). The absolute number of deaths due to communicable diseases declined from 263 in 1856 to 81 in 1950 (41% of all deaths in 1856 to 6% in 1950), whereas the number of deaths due to non-communicable diseases increased from 366 to 1,109 (57% to 85%). The absolute number of deaths due to injuries slightly increased from 12 in 1856 to 113 in 1950 (2% to 9%, Fig 1).

Trends in age- and sex-specific TB mortality in Bern 1871–1920 show that mortality was higher in the first four decades (1871–1910) than in the last (1911–1920, S3 Fig). For both sexes, TB mortality was high from infancy to early childhood (0–4 years), declined in later childhood (5–14 years) and peaked for men at 40–49 years and for women at 30–39 years. In women, TB mortality rose again at the age of 60 years and above.

### Living Conditions and TB Mortality

According to the housing survey from 1896, crowding was higher in the Black Quarter (2.2 persons per room) than in the city centre (1.4 persons per room [range 1.2–1.5]) and in the city outskirts (1.4 persons per room [range1.1–1.8]). Indoor airspace was also lower in the Black Quarter (18 m^3^/person) than in the city centre (38.5 m^3^/person [range 32–44] and the outskirts (31.8 m^3^/person [range 20–45]; Table 1). In the Black Quarter, 77% of houses had no in-door toilets, 74% had no running water and 11% were judged to be dark and humid [19].

**Table 1:** Living conditions in the ten quarters of the city of Bern around 1900 [19], comparing the Black Quarter, the quarters of the city centre (excluding Black quarter), and the city outskirts.

Characteristic	Black Quarter	City centre ^1^	City outskirts ^1^
	(n = 1)	(n = 4)	(n = 5)
Rooms per apartment, n	1.8	3.3 (2.8–4)	3.7 (2.5–4.9)
People per room, n	2.2	1.4 (1.2–1.5)	1.4 (1.1–1.8)
Airspace per apartment and person, m3	18	38.5 (32–44)	31.8 (20–45)
Airspace per person and sleeping room, m3	16	26.5 (25–29)	20 (16–22.8)
Apartments with separate living rooms, %	11.6	24.9 (18.5–27.9)	29.7 (19.9–42.6)
Apartments using rooms as sleeping and living room, %	50.2	26.3 (24.8–31)	25.1 (16.2–40.1)
Rooms without sun light ^2^, %	45.7	53.1 (45.4–56.3)	11.7 (7.7–18.1)
Rooms without ventilation ^2^, %	19	36.5 (9–161)	0.6 (0–6.3)
Apartments qualified as in “good” shape ^2^, %	4.9	15.4 (8–23.4)	25.2 (8.7–41.5)
Apartment qualified as in “bad” shape ^2^, %	45.3	24.9 (14–30.9)	16.6 (11.2–28.2)
Upper class people per 100 apartments ^2^, %	9.2	25.3 (16.9–30.9)	18.4 (8.1–24.7)
Lower class people per 100 apartments ^2^, %	70.8	37.8 (31.9–42.3)	55.3 (44.5–70.6)

^**1**^ median (minimum-maximum)

^**2**^ as judged by the statisticians of the housing survey

**Fig 2:**
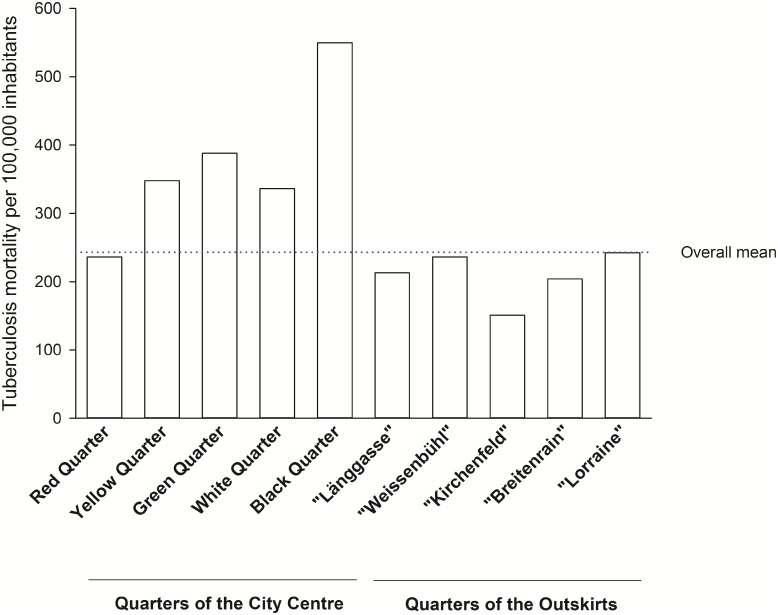
TB mortality by quarter in the city of Bern, Switzerland, between 1911 and 1915. Bern consisted of five quarters in the City centre including the “Black Quarter” (named after colours since the Napoleonic occupation), and five quarters in the city outskirts.

TB mortality differed between quarters. In 1911–1915, mortality was higher in the densely populated city centre (327 per 100,000), and particularly high in the Black Quarter (550 per 100,000), but lower in the outskirts (209 per 100,000, Fig 2). From 1921 to 1925, rates had dropped to 280 per 100,000 in the Black Quarter, 183 per 100,000 in the city centre, and 147 per 100,000 in the outskirts. The density map confirms that TB deaths in Bern were unequally distributed across quarters: they clustered in the city centre and particularly in the Black Quarter (S4 Fig).

**Fig 3:**
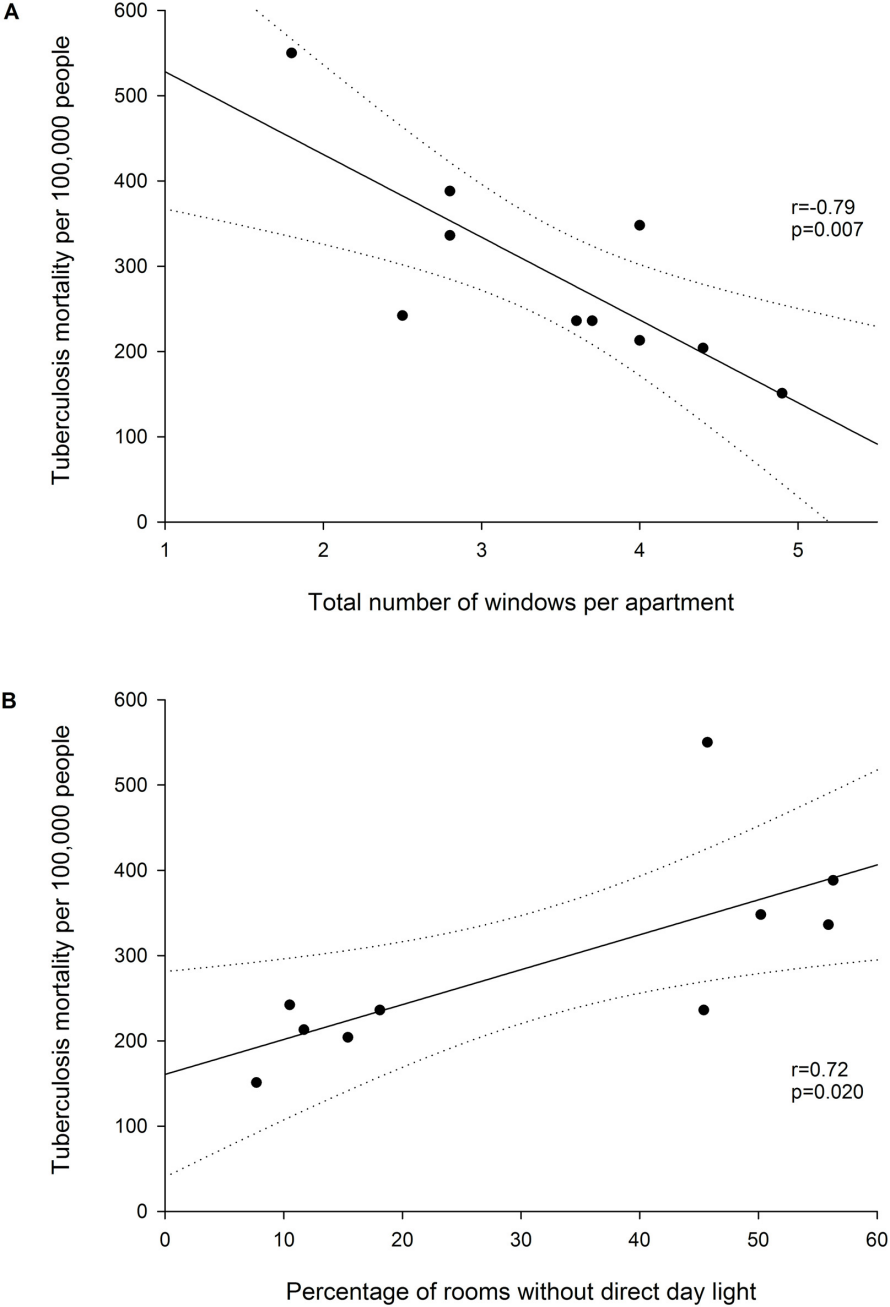
Correlation between TB mortality according the total number of windows per apartment (Fig A) and the percentage of rooms without direct day light (Fig B) in the ten quarters of Bern, 1911–1915.

TB mortality correlated positively with the number of persons per room (r = 0.69, p = 0.026, S5 Fig), with the percentage of rooms that did not have direct day light (correlation coefficient r = 0.72, p = 0.020, Fig 3A), and negatively with the number of windows per apartment (r = -0.79, p = 0.007, Fig 3B). TB mortality was also correlated with population density in the quarters (r = 0.77, p = 0.014), number of houses per km^2^ in the quarters (r = 0.81, p = 0.005), percentage of apartments in which people lived and slept in the same room (r = -0.84, p = 0.002; S6–S8 Figs).

### TB Mortality and Public Health Measures

**Fig 4:**
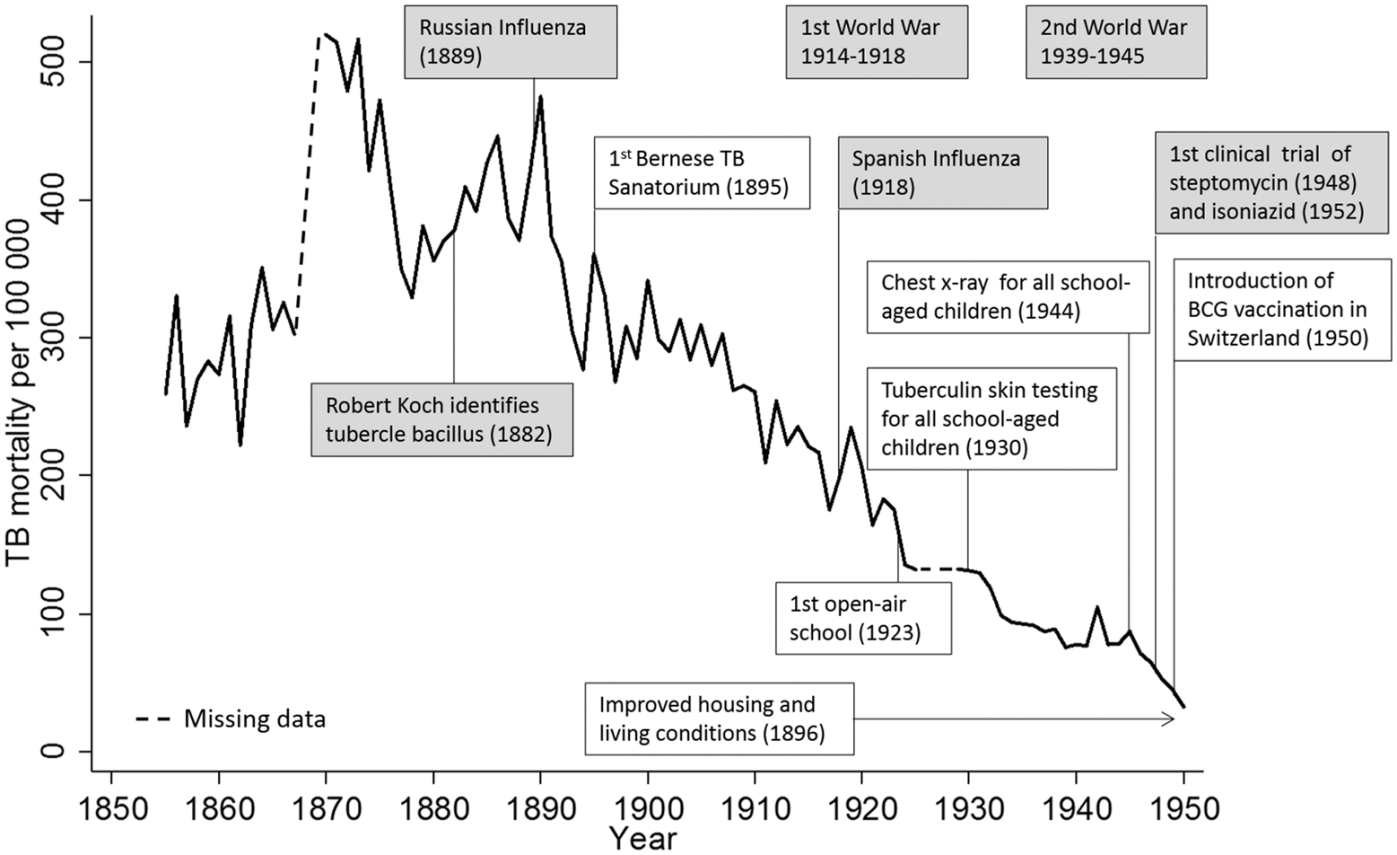
Trends in TB mortality in the city of Bern, Switzerland, between1856 to 1950, in relation to important events for TB control. Boxes in grey show worldwide events.

Fig 4 summarises the introduction of measures that are relevant to the control of TB and TB mortality in Bern. In 1895, radiography to diagnose the disease was introduced, and the first public sanatorium in Switzerland was opened outside the city of Bern (Heiligenschwendi) to isolate infectious patients. The sanatorium initially offered 50 beds, but reached its maximum capacity with 300 beds in 1950. At the sanatorium, more than 4,000 TB patients were treated between 1895–1904 and 9,000 between 1915–1924, but the number of patients steadily declined thereafter [25]. Living conditions were more favourable in the new residential areas built in the outskirts of the city in 1896. After the 1896 housing survey, some houses with poor living conditions were replaced by modern houses in the city centre, but conditions did not improve much in the Black Quarter until 1911, when a building cooperative was founded. The cooperative renovated apartments in several waves, installing with running water, kitchen, toilets and showers or baths. In the Black Quarter, some rows of dilapidated houses were demolished and replaced. In 1923 the first open-air school for undernourished children at high risk of TB opened as an alternative to sanatoria. At these schools, TB-exposed children were separated from their families with a TB case to reduce the risk of active disease and TB transmission. Other relevant measures included the introduction of systematic tuberculin skin testing of school children (1930), and of chest radiography screening (1944). Specific anti-TB drugs became available only after 1950 (Fig 4 and S1 Table).

### TB Mortality in Bern Compared to Other Regions in the Northern Hemisphere

TB mortality also decreased, for example, in the Swiss city of Zürich, in England and Wales, Germany, and the city of New York during the same time period as in the city of Bern. The annual decline ranged from 0.68% (England and Wales) to 1.74% (New York), with a good model fit for all regions (R squared 0.86–0.97; S9 Fig). The annual decline was similar in Bern compared to Zürich and Switzerland. Of note, TB mortality peaked in all those regions during the influenza epidemic in 1918.

## Discussion

TB mortality in Bern declined from 330 per 100,000 in 1856 to 33 per 100,000 in 1950, long before effective anti-TB drugs were introduced in the 1950s. The decline of TB mortality coincided with improved housing conditions in the newly built outskirts followed by renovation or replacement of buildings in the city centre and the Black Quarter by a building association. During the same period the population increased substantially and the burden of disease shifted from communicable to non-communicable diseases. The epidemiologic transition that took place in Bern is also reflected in the decline of infant mortality, a widely used indicator of development [26]. Such epidemiologic transitions continue to be observed to the present day, and are typical for many middle-income countries today [26].

We found strong associations between TB mortality and living conditions in the different quarters of Bern, including correlations between mortality and the number of people per room, percentage of rooms without direct sun light, number of windows per apartment, and population density, which reflect the socioeconomic conditions at that time. These results are consistent with observations from Paris in the 19^th^ century, which showed that the average number of windows per person was associated with TB mortality [27, 28]. Improvements of socioeconomic conditions have also been shown to be related to the decline in the TB mortality in England and Wales [9, 29–31]. In Hamburg, Germany, a higher household income was associated with a decline in the TB mortality [28, 32]. In Bern and elsewhere, the improvements of housing and sanitary conditions over time (e.g., access to running water, installation of toilets in the houses) were further reflected in the decline of mortality due to typhoid fever, which was associated to spread through contaminated water and poor hygiene. Typhoid fever and cholera have previously been shown to be strongly associated with poverty and other socio-economic factors [9, 33, 34]. The improvements of the living conditions in Bern were facilitated by the political commitment as evidenced by public health leadership of the local government [10, 13, 16]. This is in contrast to the neighbouring city of Fribourg (sharing common historic roots with Bern), where, due to lack of political will and economic resources, no measures against the poor sanitary and housing conditions were taken until the middle of the 20th century [35]. As a probable consequence, TB mortality in the city of Fribourg remained one of the highest in the country until 1930 [36].

The major decline in TB mortality took place long before effective TB drugs combination therapy became available in the 1950s, and factors other than drugs need to be considered to explain the decline [3, 4, 6, 9, 37, 38]. Our results suggest that a combination of improvements over time, including improved housing conditions and particularly ventilation, screening for TB at schools, isolation of TB cases from the general population in TB sanatoria and open-air schools may all have contributed to the decline of TB in the city of Bern. The decline in mortality may also be partially explained by the natural behaviour of an epidemic, reflecting the decline of epidemics on very long time scales [37]. Other factors might also have influenced the decline, such as the natural selection of genetically more resistant human populations [9]. However, it is merely impossible to disentangle all the different factors potentially contributing to the TB mortality decline [3].

Similar declines in TB mortality were also observed in other regions in the Northern hemisphere [1, 3, 4, 6, 21–23, 38, 39]. In all European countries, TB mortality decreased steadily, but peaked 1918 due to the influenza epidemic [40].

Analyses of historic mortality data have several limitations. In particular, the ascertainment of TB-related deaths can be difficult. It is unknown to what extent deaths were truly due to TB because the causative agent, *Mycobacterium tuberculosis*, was identified by Robert Koch only in 1882 [41]. Bacteriological confirmation was therefore not available in the earlier years of our study [11]. However, the cause of death was filled out by the medical doctors on a specific death card, and for most deaths the cause was established through official autopsies [10].

In conclusion, improved living conditions and public health measures likely led to the decline in TB mortality in Bern before effective treatment options became widely available. Future research by modelling the relative impact of the various co-factors based on historic data may improve our understanding of the contemporary TB epidemics, and inform predictions and TB control strategies.

## Supporting Information

S1 Fig Historic map of the city of Bern with its ten quarters: city centre (1–5) including the Black Quarter (5), and the city outskirts (6–10). Quarters were named after colours since the Napoleonic occupation [19].
(TIF)

S2 Fig Decline in TB mortality compared to the decline in typhoid fever mortality per 100,000 population, and the infant mortality (children <1 year) per 1,000 live births in the city of Bern, Switzerland (average 2-years mortality). Regression lines were calculated based on the following time periods: for TB and infant mortality 1880–1945, for typhoid fever 1856–1945. The decline in mortality was presented as annual decline in percentage, and the fit of the regression lines as R squared.
(TIF)

S3 Fig Cross-sectional TB mortality rates per 100,000 population for men (Fig A) and women (Fig B) by age in Bern between 1871 and 1925. (TIFF)

S4 Fig Density plot of TB deaths by residency in the city of Bern between 1920 and 1935, with a map insert showing the population density in the ten quarters. The colours correspond to the number of deaths found within a 300 m radius (0.282 km^2^). The light grey line denotes the border between residential areas and the uninhabited countryside, and the dark grey line borders of the quarters. The river Aare is shown in blue. Numbers on the map correspond to the ten quarters of Bern (see also S1 Fig): city centre (1–5) including the Black Quarter (5), and the city outskirts (6–10).
(TIFF)

S5 Fig Correlations between the TB mortality according mean number of people per room in the ten quarters of Bern, 1911–1930. (TIF)

S6 Fig Correlations between the TB mortality according population per km^2^ in the ten quarters of Bern, 1911–1930. (TIF)

S7 Fig Correlations between the TB mortality and number of houses per km^2^ in the ten quarters of Bern, 1911–1930. (TIF)

S8 Fig Correlations between TB mortality and the percentage of houses with separate rooms for living and sleeping, and shared living/sleeping rooms in the ten quarters of Bern, 1911–1915. (TIF)

S9 Fig Decline in TB mortality over the last 150 years in the city of Bern, compared to other cities and countries of the Northern Hemisphere. The nation-wide data from Switzerland include the data from the cities of Bern and Zürich. Regression lines were calculated based on the following time periods: for Bern from 1880–1945, for Switzerland from 1900–1945, for Zürich from 1893–1933, for Germany from 1892–1945, for England and Wales from 1852–1923 and for New York from 1881–1945. The decline in mortality was presented as annual decline in percentage, and the fit of the regression lines as R squared.
(TIF)

S1 File Dataset underlying the findings in the manuscript. (XLSX)

S1 Table Important events detailed from 1900 to 1944. Data sources: Refs. [1–3].
(DOCX)
