# Supplementary figures and images for: Tuberculosis Mortality and Living Conditions in Bern, Switzerland, 1856-1950
Source: PLoS One. 2016 Feb 16;11(2):e0149195. doi: 10.1371/journal.pone.0149195 (PMC4755532; doi:10.1371/journal.pone.0149195)

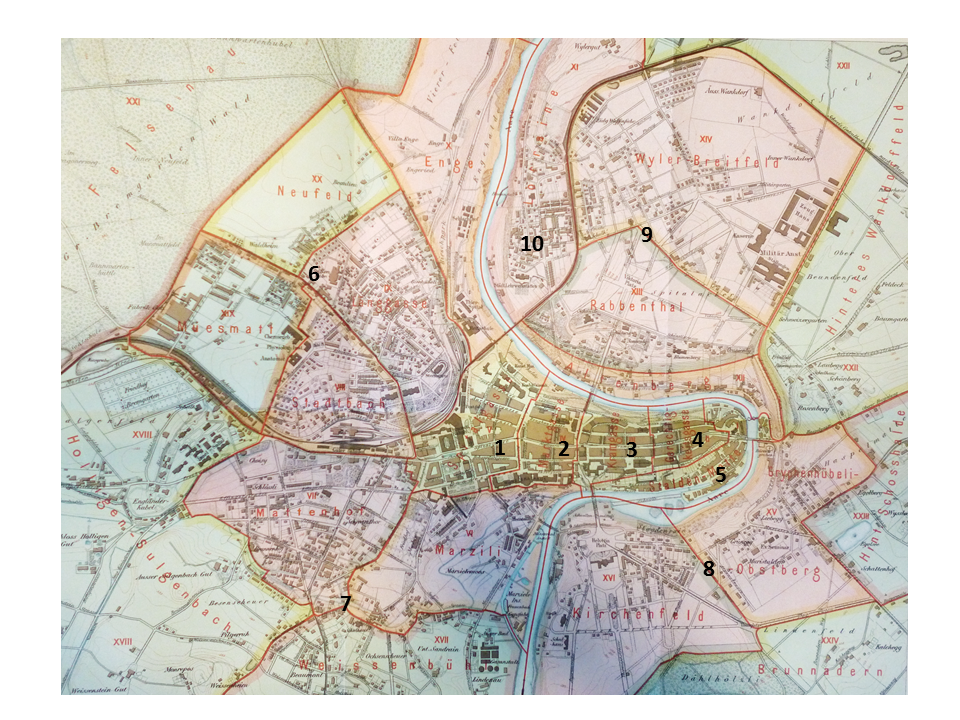

Supplement: S1 Fig — Quarters were named after colours since the Napoleonic occupation [19]. (TIF) [file pone.0149195.s001.tif]

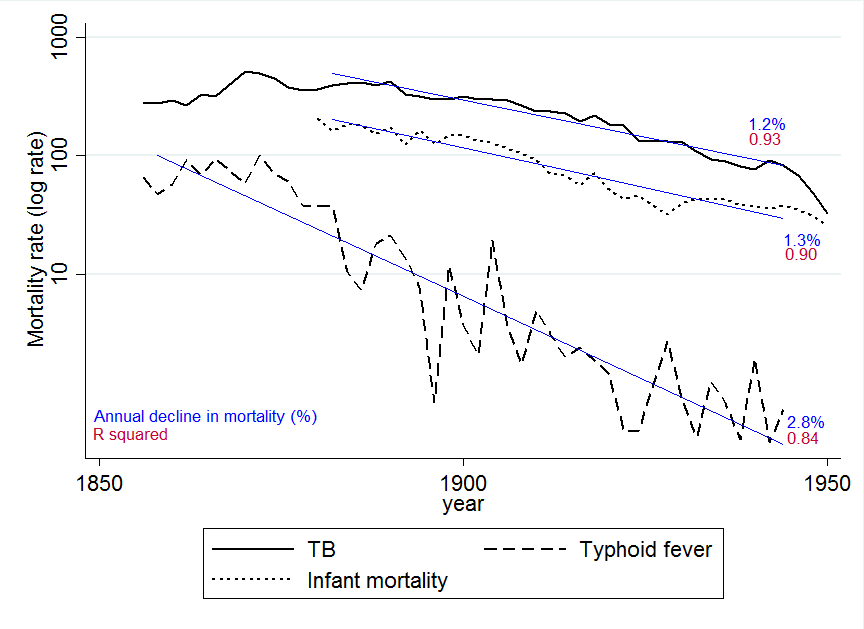

Supplement: S2 Fig — Regression lines were calculated based on the following time periods: for TB and infant mortality 1880–1945, for typhoid fever 1856–1945. The decline in mortality was presented as annual decline in percentage, and the fit of the regression lines as R squared. (TIF) [file pone.0149195.s002.tif]

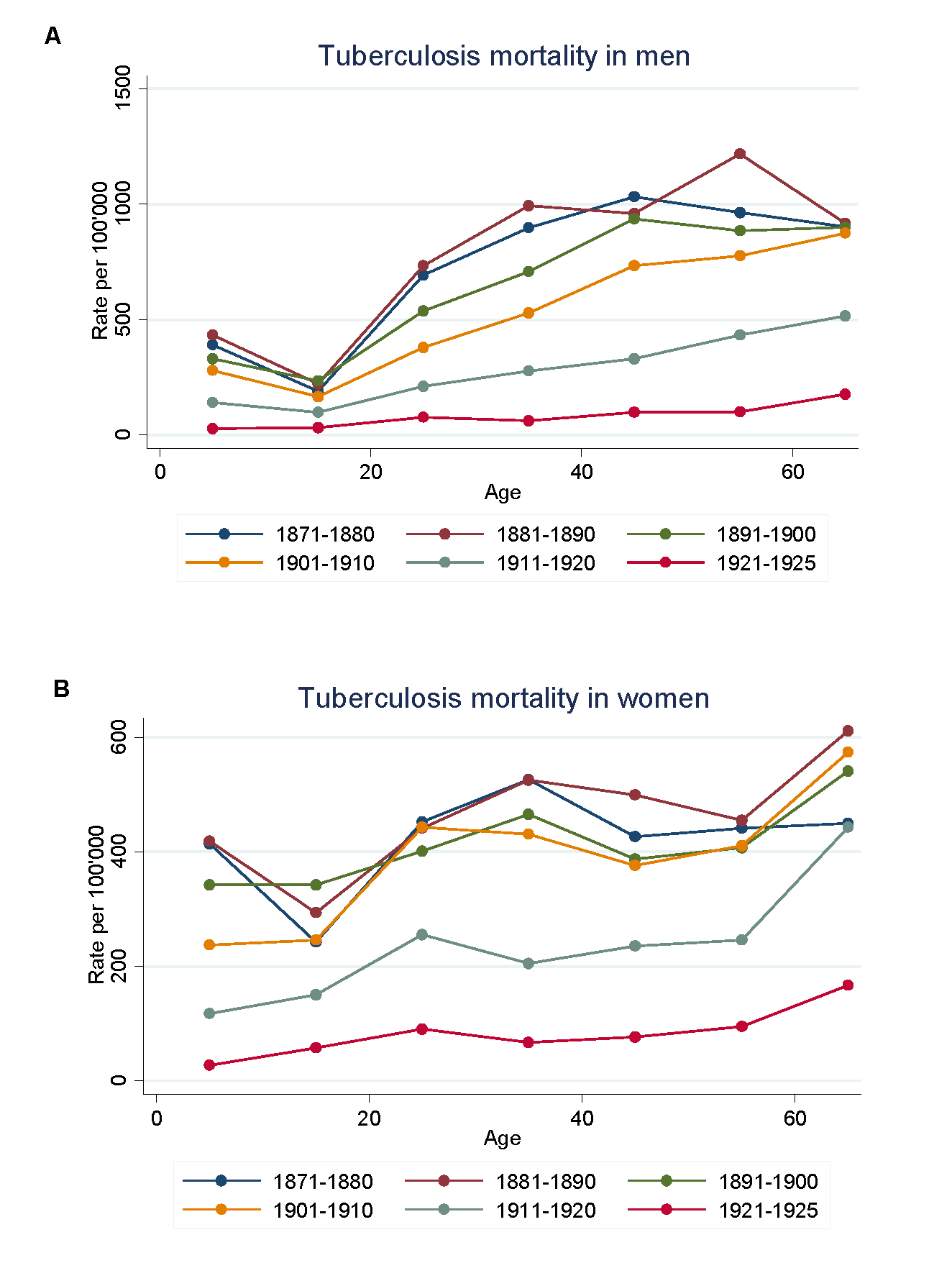

Supplement: S3 Fig — (TIFF) [file pone.0149195.s003.tiff]

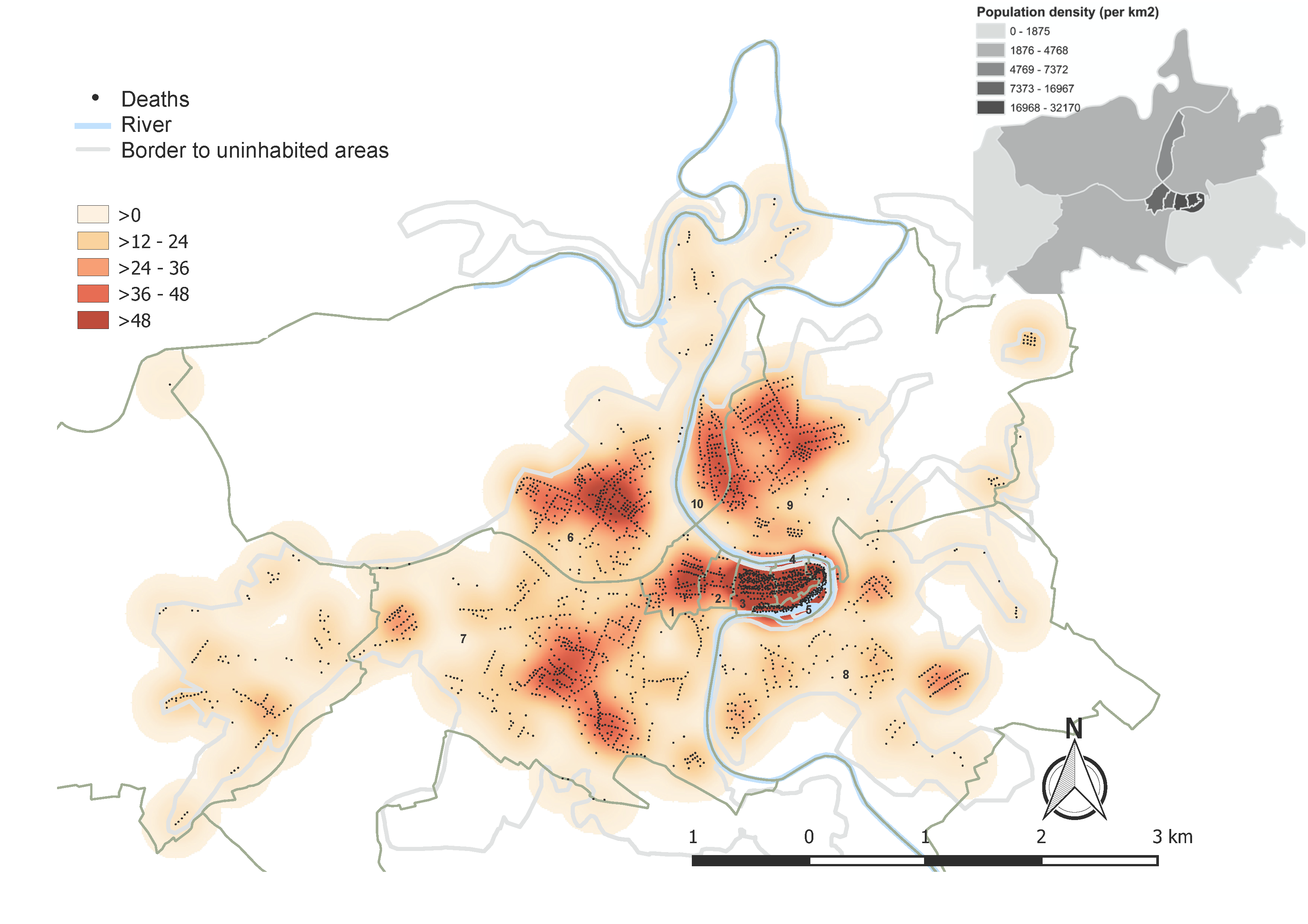

Supplement: S4 Fig — The colours correspond to the number of deaths found within a 300 m radius (0.282 km2). The light grey line denotes the border between residential areas and the uninhabited countryside, and the dark grey line borders of the quarters. The river Aare is shown in blue. Numbers on the map correspond to the ten quarters of Bern (see also S1 Fig): city centre (1–5) including the Black Quarter (5), and the city outskirts (6–10). (TIFF) [file pone.0149195.s004.tiff]

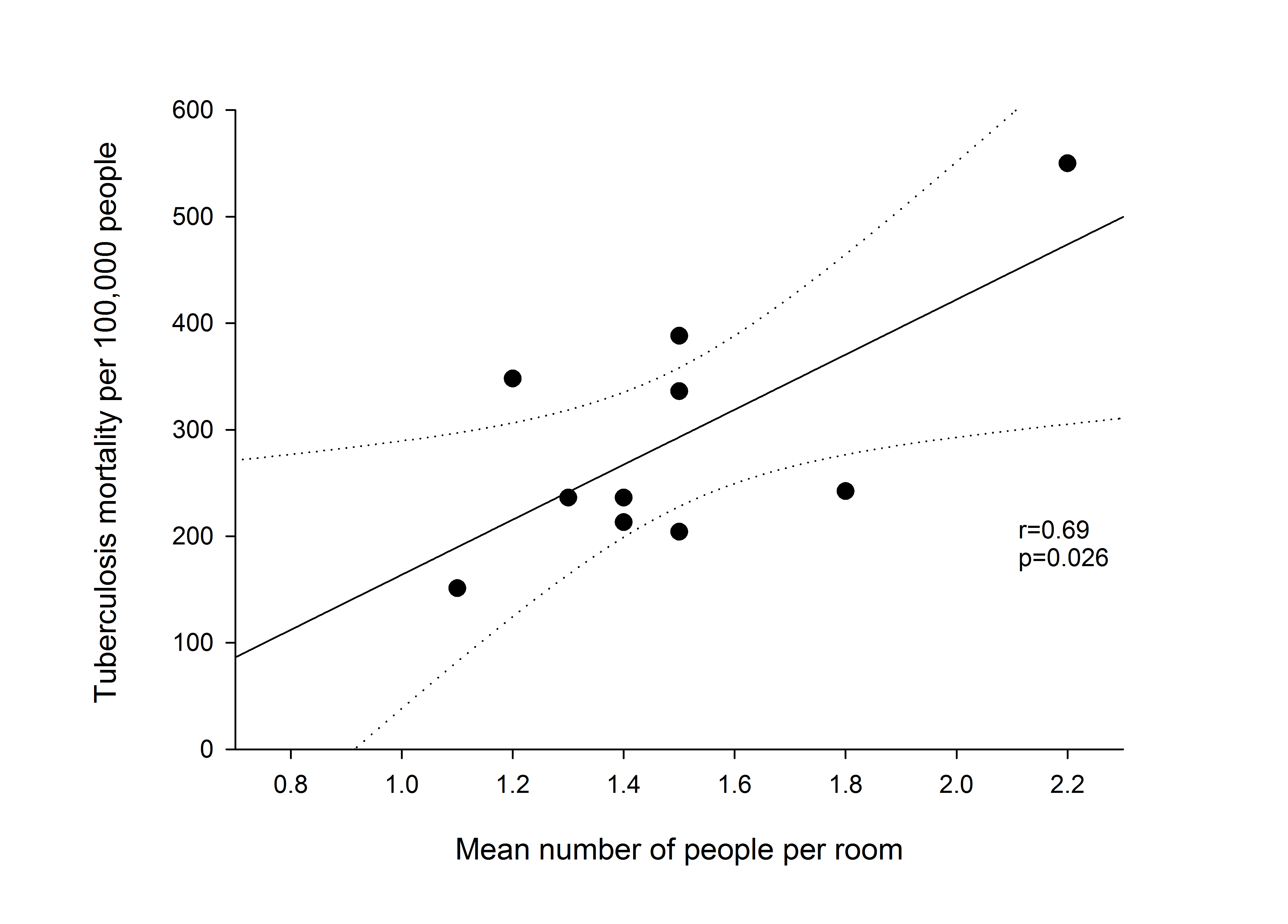

Supplement: S5 Fig — (TIF) [file pone.0149195.s005.tif]

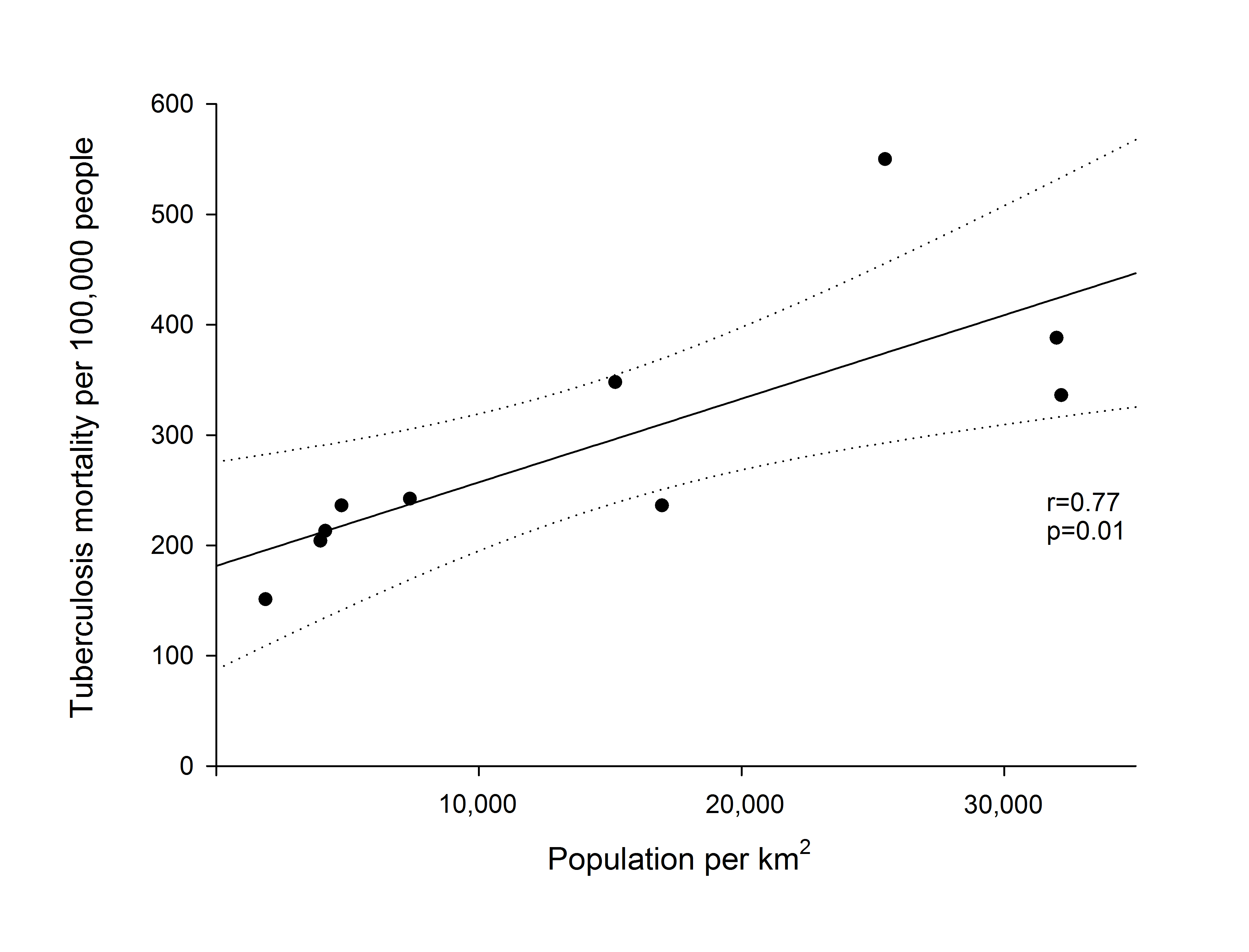

Supplement: S6 Fig — (TIF) [file pone.0149195.s006.tif]

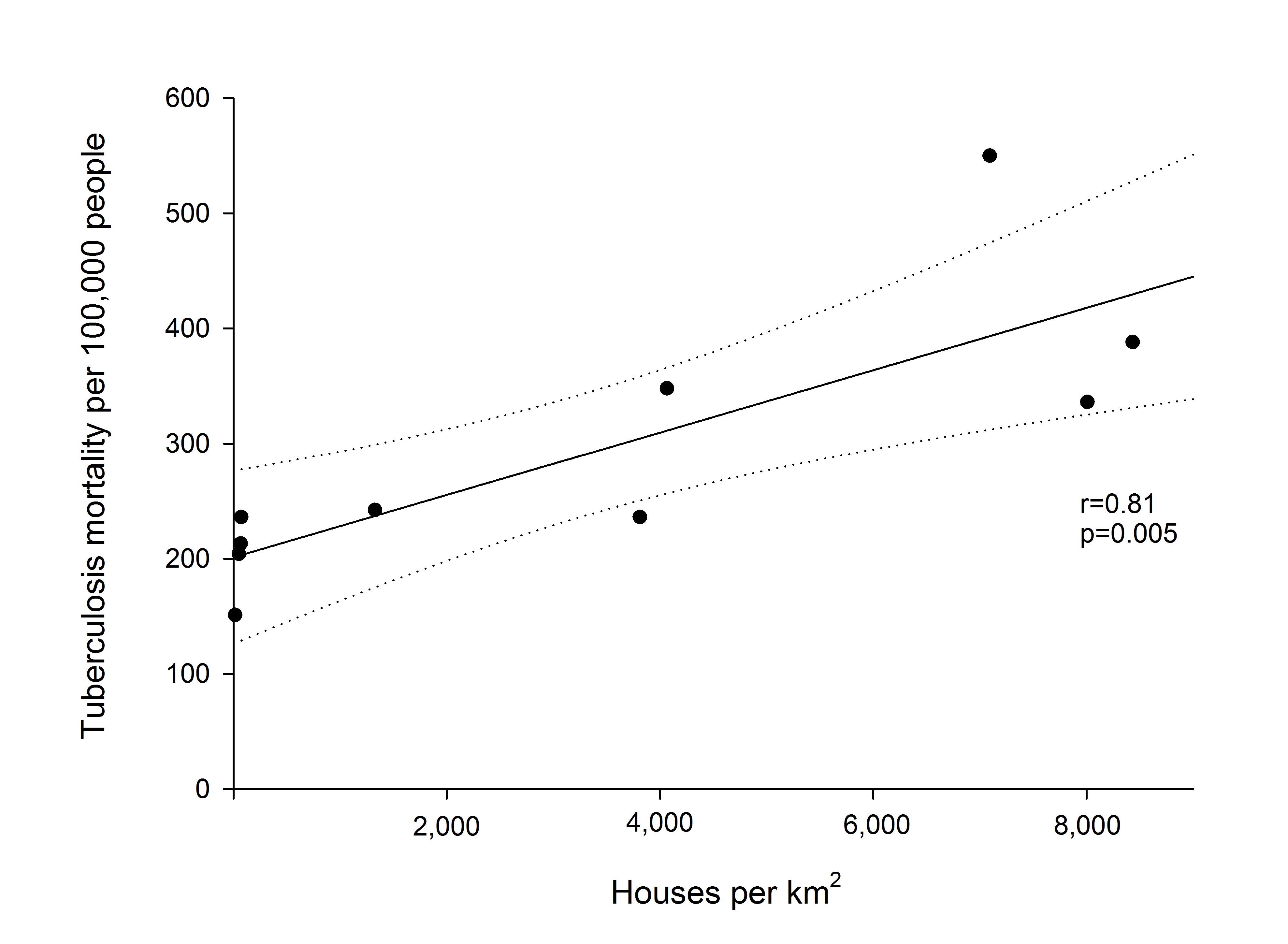

Supplement: S7 Fig — (TIF) [file pone.0149195.s007.tif]

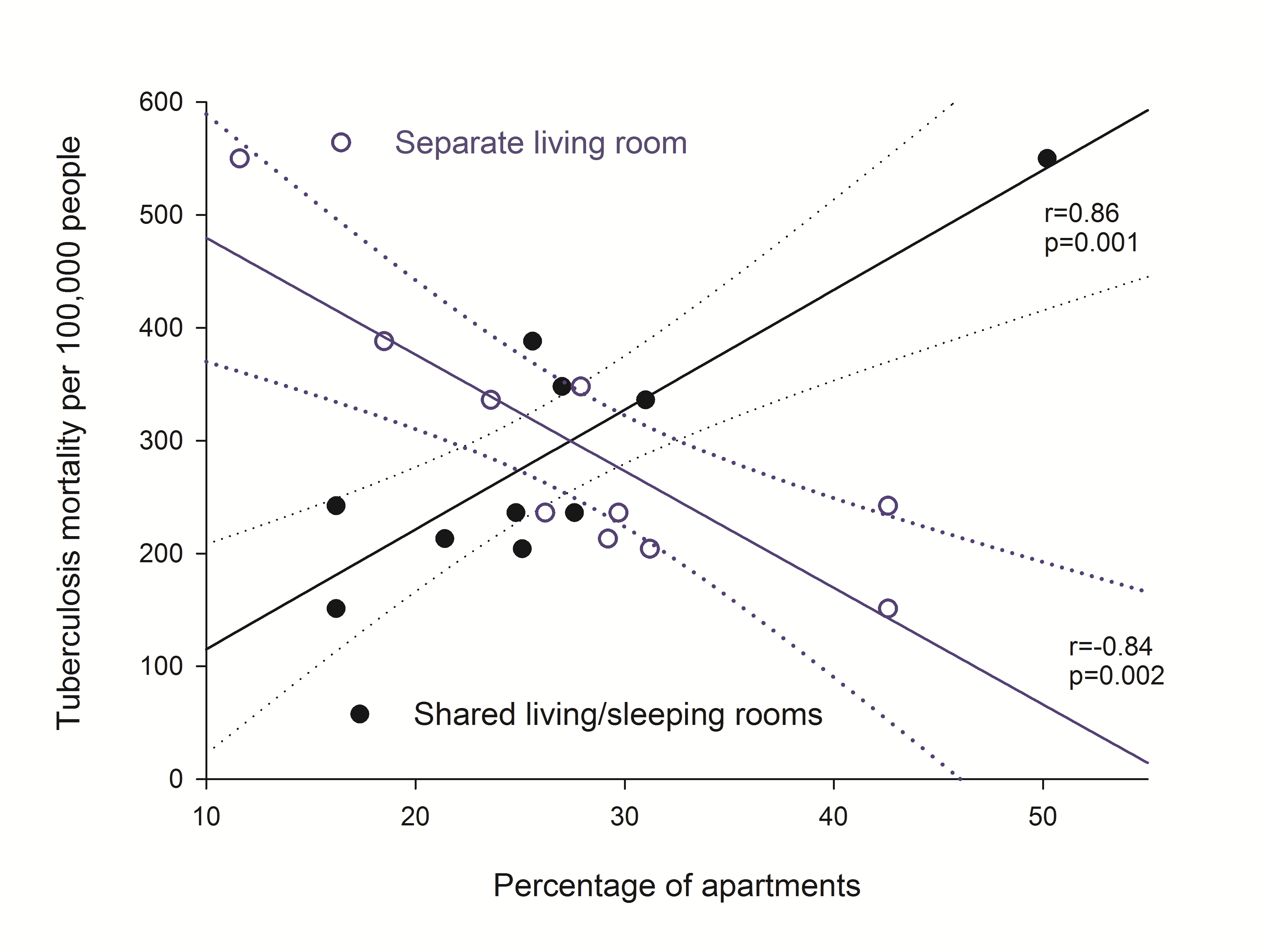

Supplement: S8 Fig — (TIF) [file pone.0149195.s008.tif]

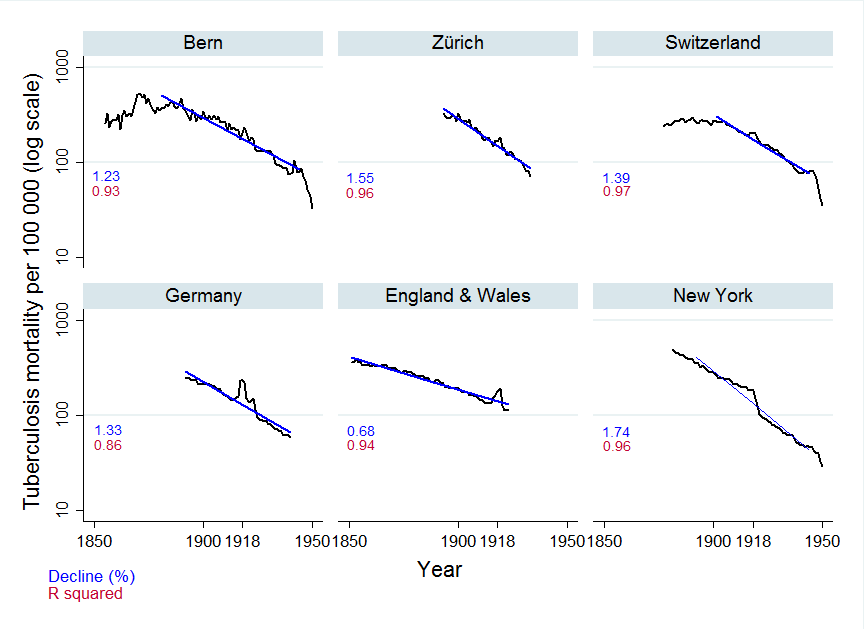

Supplement: S9 Fig — The nation-wide data from Switzerland include the data from the cities of Bern and Zürich. Regression lines were calculated based on the following time periods: for Bern from 1880–1945, for Switzerland from 1900–1945, for Zürich from 1893–1933, for Germany from 1892–1945, for England and Wales from 1852–1923 and for New York from 1881–1945. The decline in mortality was presented as annual decline in percentage, and the fit of the regression lines as R squared. (TIF) [file pone.0149195.s009.tif]
