# Supplementary material for: Tuberculosis Mortality and Living Conditions in Bern, Switzerland, 1856-1950
Source: PLoS One. 2016 Feb 16;11(2):e0149195. doi: 10.1371/journal.pone.0149195 (PMC4755532; doi:10.1371/journal.pone.0149195)
Supplement: S1 Table — Data sources: Refs. [1–3]. (DOCX) [file pone.0149195.s011.docx]

**Table S1.** Important events detailed from 1900 to 1944. Data sources: Refs. (1-3).

| **Year** | **Event** |
| --- | --- |
| 19^th^ century | Health policy means disease policy. Communicable diseases like typhus, cholera and TB were associated with dirt, sickening smell and the bad living conditions. In Bern, the Black Quarter was mostly affected by these diseases. |
| 2nd half of the 19th century | Hygiene measures (hygiene and housekeeping) were introduced by doctors and natural scientists to address the problems. The industrialisation resulted into a six-fold increase of the population and the housing conditions were described as precarious. |
| 1880s | Building boom and rapid urbanisation. The lower class lived in precarious conditions. There was no functioning sewage water system, and the fresh water supply was not consequently controlled, which made Bern an ideal ground for epidemics. |
| 1881-1890 | **Dr. Wilhelm Ost** (1853-1922), MD, police doctor of Bern since 1896, and was one of the founders of the non-profit building cooperative. He was strongly committed to public health care and was for example co-founder of the TB sanatorium “Heiligenschwendi”. In 1907, he was elected in the city council as a member of the Social Party, and was also a member of the cantonal medical college (“Sanitätskollegiums”). He died in 1922, and donated a considerable part of his inheritance to the city to improve the living conditions in the Old Town.  Dr. Ost noticed that the Black Quarter had a two-fold higher TB mortality compared to other quarters. |
| 1889 | Housing storage in the city. Many families found no accommodation and had to stay in the forest near by the city. |
| 1890-96 | The Outskirts “Wyler” and “Ausserholligen” were built (182 appartments). |
| 1895 | Founding of the Health Care association  - 1899 other organisation joined  - 1909 non-profit building cooperative was founded. |
| 1895 | First Berner TB Sanatorium in Heiligeschwendi opened outside of Bern. |
| 1896 | First housing survey („Wohnungsenquête“). |
| ~1910 | Infantile and mother courses. The mothers were tested after birth on TB and if sputum was available, it was tested bacteriological. |
| 1911 | Founding of the building cooperative: The building cooperative bought old buildings to rebuild them. The founders established the renovation of the quarters with a high TB mortality (city centre, Black Quarter). The mortality statistic and the housing survey (“Wohnungenquete” (1896”) showed that the Black Quarter had the highest TB mortality and the worst living conditions. Reconstructions at several sites (“Badgasse”, “Bubenberg Rain”, “Gerbergasse” and “Mattenenge”). Between 1915-1932, there were five waves of renovations:  1st wave: basic toilets and kitchens (1916-17)  2nd wave: Frickbad (1923-24)  3th wave: wash basins in bedrooms (1926-27)  4th wave: toilets equipped with showers (1927-28)  5th wave: refurbishment of rooms (1931-32) |
| 1913 | The city council reorganized the school medical care for children and adolescents. From now on, a dedicated school doctor was taking care of children at schools. |
| 1914-18 | 1st World War: The effects of poverty due the war had influence of children’s health. For example: growth retardation, deficiency symptoms due to food rationing. Children were given nutritional supplements and vitamins B, C and D. |
| 1917 | Families housed in barracks and schools because of housing shortage. |
| 1918 | Influenza epidemic (“Spanish influenza”). |
| 1919 | The first time that the cantons and national government decided to subsidise the housing conditions (first round of subsidies). |
| 1922 | Death of Dr. Wilhelm Ost. He donated a large part of his heritage to the building cooperative. |
| 1923 | First open-air school at the Elfenau opened for undernourished children and children at a higher risk of TB. |
| 1929 | International finance crisis |
| 1929 | Public health law to control TB. 1930 introduced in the city of Bern. Tuberculin skin test for all school-aged children (grade 1, 5, and 9) Grades). Skin test positive children were referred to a hospital (“Ziegler Spital”) for further investigations. |
| 1931 | The building cooperative advertised a competition for the renovation of several streets. First streets in the Black Quarter were: ”Nydegghöfli and Matte Enge”. The Bernese homeland security made an objection. 1939 the building cooperative adapted the plans according the criticism of the homeland security. Construction work was postponed during the second World War. |
| 1939-1945 | 2^nd^ World War |
| 1942 | Second round of subsidies of housing to improve housing conditions |
| 1944 | Introduction of systematic chest X-ray screening for all school-aged children (grade 1, 5 and 9). |
| 1948 | First clinical trial of Streptomycin as an antibiotic against TB. First effective treatment for TB. |
| 1950 | BCG vaccination Switzerland introduced. |
| 1952 | First clinical trial of isoniazid (isonicotinoyl hydrazide). Effective treatment for TB. |

**References**

1. Hofmann M, Schnell D, Meister H, Kehrli M. Licht und Luft: Gemeinnützige Baugenossenschaft Bern aus Anlass ihres 100-jährigen Bestehens. 2011.

2. Nepfer M. Die Wohnungsfrage in der Stadt Bern 1888-1950: Regulierung des Wohnverhaltens durch sozialen Wohnungsbau? 1996.

3. Tschumper A, Ackermann U, Vuille JC, von Albertini M. 1913-2013 Vom Schularztamt zum Gesundheitsdienst: Festschrift anlässlich des 100-Jahr Jubiläums des Gesundheitsdienstes. 2013.
